# Predicting mechanical complications in proximal femoral nailing for elderly patients: a radiological scoring system based on a single-centre retrospective cohort with 586 cases

**DOI:** 10.1007/s00068-025-02850-6

**Published:** 2025-04-12

**Authors:** Cafer Özgür Hançerli, Halil Büyükdoğan

**Affiliations:** 1 Bahçeşehir University, Medical Park Göztepe Hospital, Department of Orthopaedics and Traumatology, Istanbul, Turkey; 2 Health Sciences University, Istanbul Kanuni Sultan Süleyman Training and Research Hospital, Department of Orthopaedics and Traumatology, Istanbul, Turkey

**Keywords:** Proximal femoral nailing (PFN), Intertrochanteric femur fractures, Mechanical complications, Surgical scoring systems, Intraoperative optimisation

## Abstract

**Background:**

Proximal femoral nailing (PFN) is a preferred treatment for intertrochanteric femoral fractures in elderly patients due to its minimally invasive nature and early mobilisation benefits. However, mechanical complications such as implant failure, cutout, and reduction collapse remain significant challenges. This study introduces the targeted surgical score (TSS), a novel scoring system designed to predict and mitigate mechanical complications by evaluating modifiable surgical factors.

**Methods:**

A retrospective analysis of 586 patients aged 65 and older treated with PFN between 2015 and 2022 was conducted. Data on demographic characteristics, fracture classifications, and surgical parameters were collected. Radiographic assessments included tip-apex distance (TAD) and lag screw positioning for implant placement quality, medial and anterior cortical support (MCS and ACS), and fracture alignment in both AP and lateral planes for reduction quality. Each parameter was scored, resulting in a cumulative TSS ranging from 0 to 8. Logistic regression and ROC curve analysis were performed to evaluate the predictive capacity of TSS.

**Results:**

The average TSS was 4.06 ± 2.22 in the complication group and 6.14 ± 1.56 in the non-complication group (p < 0.001). A one-point increase in TSS was associated with a 44.9% reduction in complication risk (OR 0.551; p < 0.001). Independent risk factors included lag screw placement (non-central superior quadrants), inadequate cortical support in AP and lateral planes (MCS and ACS), and TAD (≥ 25 mm). The TSS demonstrated relatively good discriminative ability with an AUC of 0.768.

**Conclusion:**

TSS may aid in predicting and mitigating mechanical complications while potentially guiding surgical applications in PFN, but further prospective multicentre validation is required. While certain parameters of TSS could be considered intraoperatively, its full implementation may be more practical for postoperative risk assessment.

## Background

Fractures of the trochanteric region of the femur are one of the most common injuries that can occur with low-energy trauma in an increasingly elderly population and osteoporosis [1, 2]. These fractures are among the serious injuries that carry a high risk of morbidity and mortality and often require surgical treatment [3]. With the development of implant technology, proximal femoral nailing (PFN) is frequently preferred in surgical treatment as a minimally invasive method that offers additional advantages in terms of stability and thus allows early mobilisation [4, 5]. Despite these advantages, mechanical complications such as reduction collapse, cutout, and implant failure remain important concerns in clinical practice [6, 7].

It is known that many patient, fracture and surgical factors play a role in the occurrence of mechanical complications after PFN treatment of femoral intertrochanteric fractures [8]. In the literature, many studies address the risk factors associated with complications [9, 10]. These studies have focused on non-modifiable patient characteristics such as the degree of osteoporosis, fracture type and stability, and correctable surgical factors such as implant placement technique and reduction quality [11]. In particular, surgical parameters such as tip-apex distance, varus reduction, quadrant of lag screw placement, and fracture alignments in the anteroposterior (AP) and lateral planes have been reported to be strongly associated with the risk of complications and treatment failure [12]. However, most studies have focused on determining the risk factor most associated with mechanical complications and treatment failure [13, 14].

This study analysed non-modifiable patient-related and modifiable surgeon-related risk factors. Under the guidance of existing studies in the literature, the main objective was to evaluate the cumulative effects of many factors that may be associated with treatment failure due to mechanical complications, in addition to their independent effects [15]. Our study aims to examine the impact of surgical factors on the risk of mechanical complications by analysing the implant placement technique and fracture reduction quality in PFN application in detail. In this direction, the aim is to develop a strategic approach to reduce the surgical risk factors and to create a scoring system to predict postoperative mechanical complications and minimise treatment failure.

## Methods

### Study design and patient population

This retrospective study was performed by analysing the data of patients who underwent surgical treatment for femoral intertrochanteric fracture in a tertiary trauma centre. The study included patients operated between January 2015 and December 2022. The study was conducted with the approval of the local ethics committee and following the Declaration of Helsinki.

The study included patients aged 65 years and older who were hospitalised with a diagnosis of femoral intertrochanteric fracture and underwent a PFN. The study population consisted of patients who developed treatment failure due to mechanical complications such as cutout and implant failure in post-discharge follow-up radiographs and patients with no mechanical complications detected in follow-up radiographs at least 9 months after discharge.

### Surgical and follow-up procedure

All operations were performed in a single centre under spinal or general anaesthesia following preoperative routine anaesthesia preparations, fluoroscopically controlled closed reduction of the patients on the traction table and appropriate asepsis and antibiotic prophylaxis protocols. During the surgical procedure, a short length (200–220 mm) anti-rotation PFN system with trochanteric tip entry, lag screw-nail angle 125°, 10–12 mm diameter, was inserted with a minimally invasive method under fluoroscopy.

Early postoperative weight-bearing as tolerated was recommended for patients with stable fracture types and those whose fracture reduction and fixation were deemed stable. In contrast, patients with unstable fractures or inadequate fracture reduction and fixation were advised to follow a more protective rehabilitation approach, including a partial weight-bearing (PWB) protocol for 6–12 weeks using walkers or crutches. PWB was advised explicitly for patients with poor fracture reduction, suboptimal TAD, or an inappropriate lag screw quadrant [13]. Routine follow-up visits at 2 weeks, 6 weeks, 3 months, 6 months and 9 months after discharge were recommended.

### Exclusion criteria

Patients with pathological fractures occurring in the background of neoplastic disease, patients with polytraumas and multiple fractures, patients who did not use a traction table or underwent open reduction, patients with inappropriate radiological imaging and patients who underwent early revision surgery before discharge from the hospital were excluded from the study.

### Data collection and evaluation parameters

Patient data were obtained through the electronic patient record system. All screening and radiological measurements were made by a senior orthopedic assistant who was not included in the study via the hospital picture archiving and communication systems.

Demographic data included age, gender, and fracture side. In contrast, the age groups of the patients (65–74 years, 75–84 years, 85 years and older) were included as a separate parameter since there may be a difference in bone quality and general health conditions [16].

Parameters with the potential to affect perioperative or follow-up processes, such as American Society of Anesthesiologists (ASA) score, type of anaesthesia (spinal, general), operation waiting time (days), surgical time (minutes), discharge time (days) and body mass ındex (BMI) were also noted.

Fracture type was analysed according to AO/OTA classification and stable (31 A1.1 and A1.2)/unstable (A1.3 and above). Cortical thickness index (CTI) and canal flare index (CFI) measurements were performed to observe differences in bone quality and morphological characteristics of the proximal femur [17–19].

**Fig. 1:**
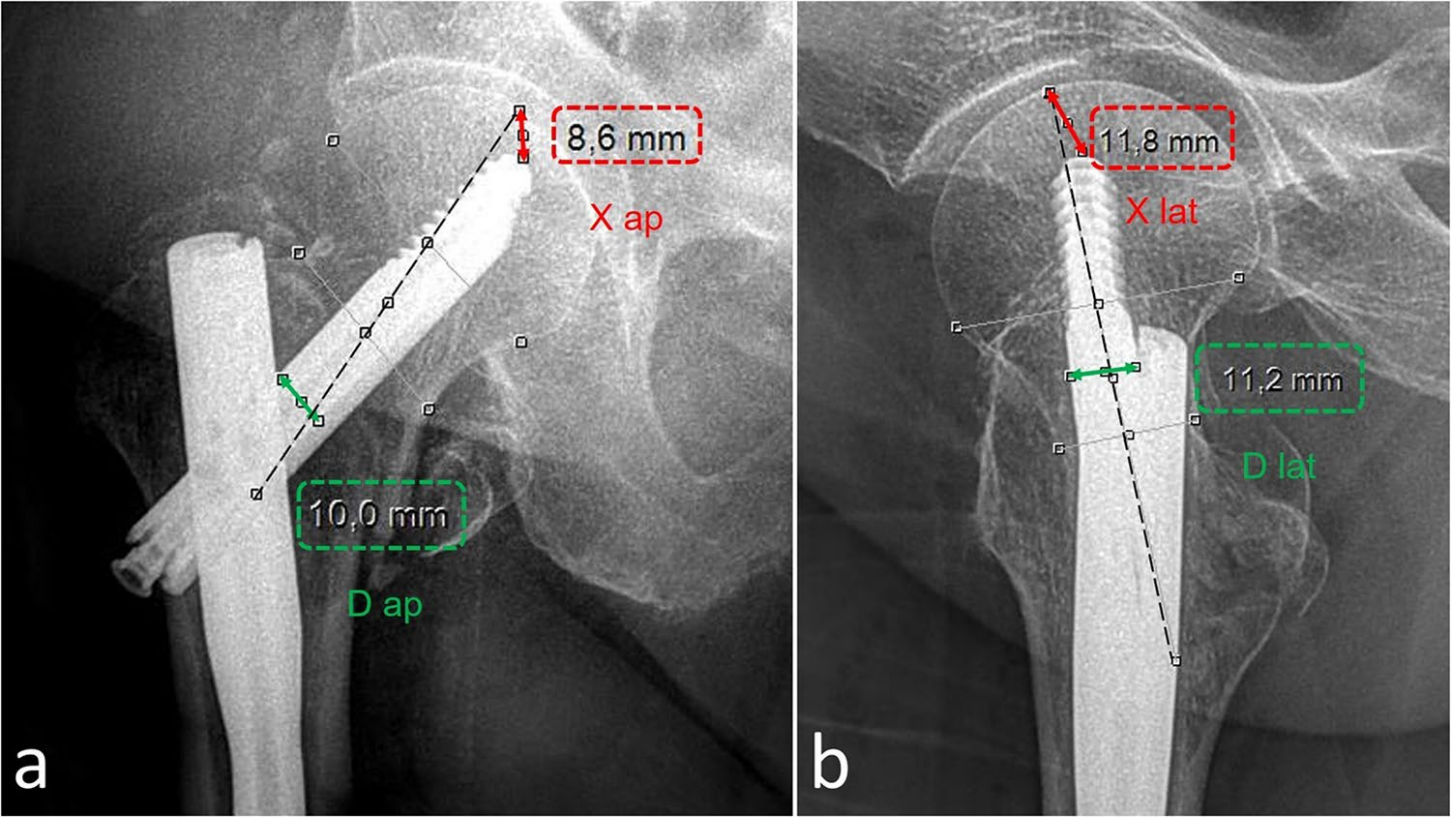
Measurement and calculation of the Tip-Apex Distance. TAD = (X-ap x D-true/D-ap) + (X-lat x D-true/D-lat). Measurements on AP radiograph (**a**). Measurements on lateral radiograph (**b**)

**Fig. 2:**
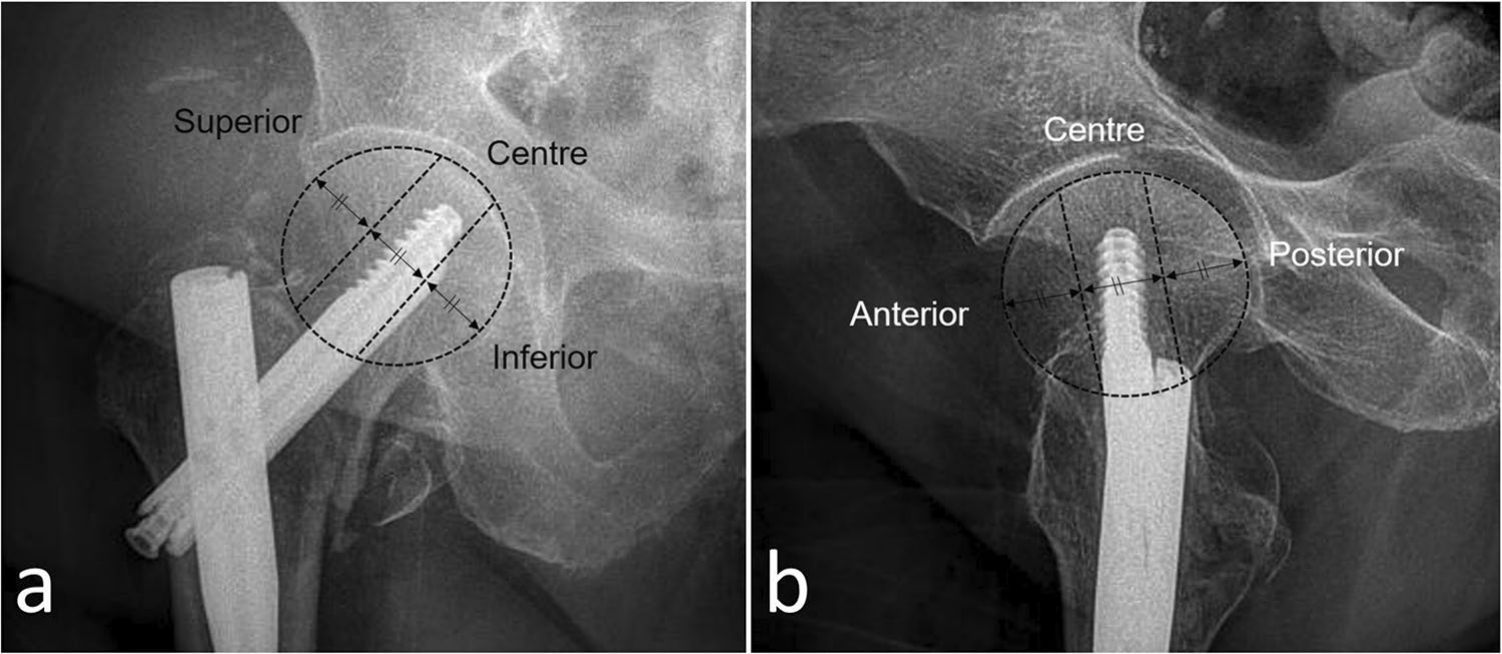
Determination of the lag screw quadrant. The femoral head was divided into three equal parts in AP and lateral planes in the direction of the neck axis. Superior, centre, and inferior quadrants on AP radiograph (**a**). Anterior, centre, and posterior quadrants on lateral radiograph (**b**)

**Fig. 3:**
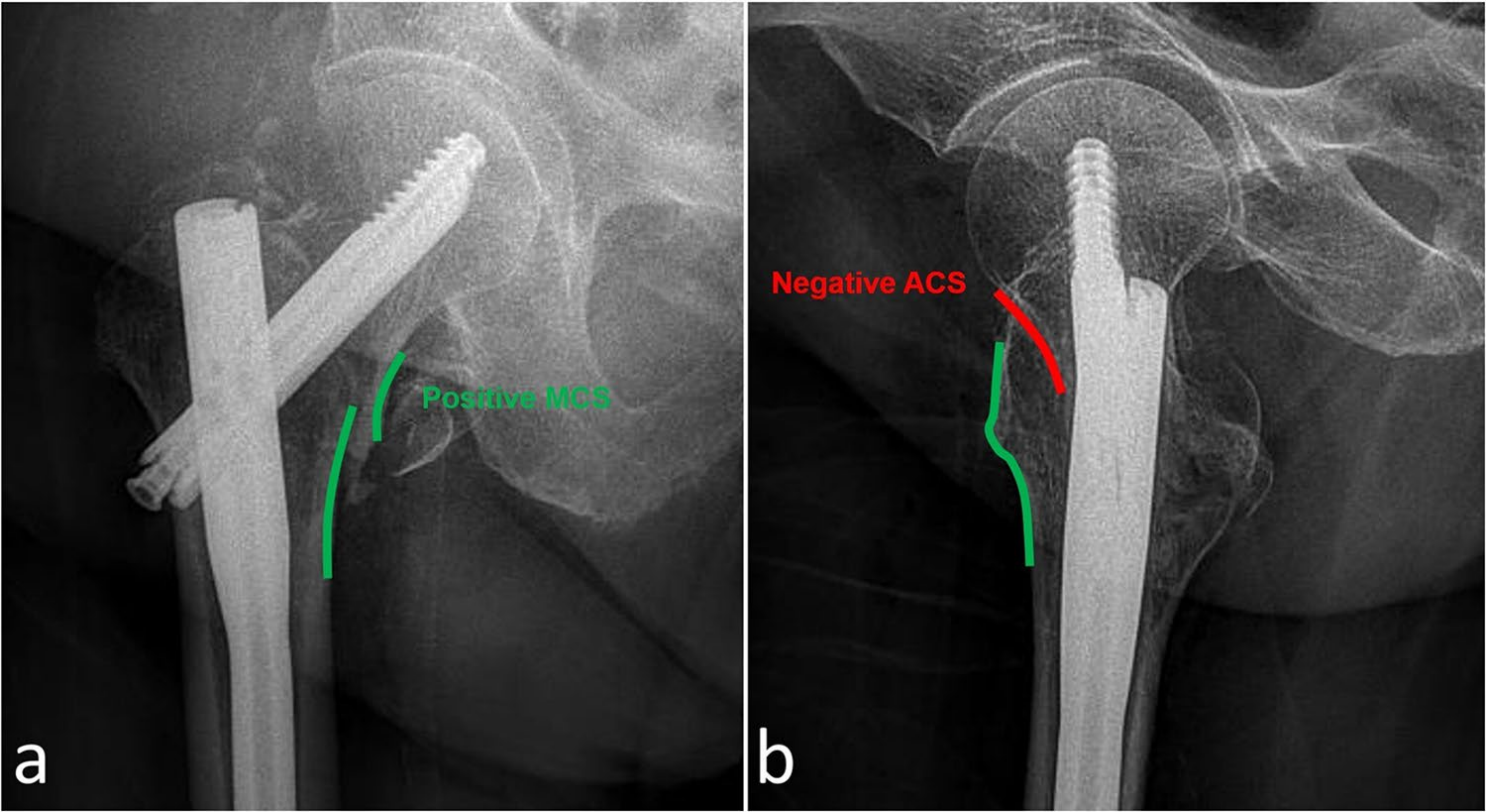
Examination of cortical support between fracture fragments to assess the reduction quality. Positive medial cortical support on AP radiograph (**a**). Negative anterior cortical support on the lateral radiograph (**b**)

**Fig. 4:**
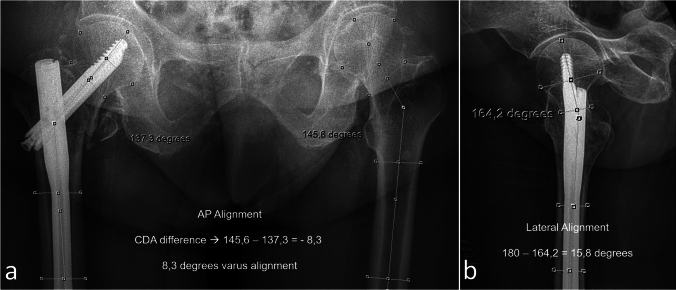
Evaluation of fracture alignment in AP and lateral planes. The difference in collo-diaphyseal angle compared to the intact hip on AP radiograph (**a**). Angulation between the diaphyseal axis and the head-neck axis in the lateral plane (**b**)

Preoperative and postoperative routine pelvic and hip X-ray images were used in radiological evaluations. The patients whose X-ray images were unsuitable were evaluated by examining the fluoroscopy images recorded during the operation. Collo-diaphyseal angle (CDA) measurements were recorded on the intact and fractured sides. The tip-apex distance (TAD) defined by Baumgartner and the location of the lag screw in the Cleveland–Bosworth quadrant (lag quadrant) were determined and noted [8, 20, 21]. In addition, the reduction quality was evaluated according to Chang’s criteria; the difference between the fractured side CDA and the intact side CDA was calculated for the appropriateness of the alignment in the AP plane. A difference between 0° and 10° (i.e., neutral or < 10° valgus alignment) was considered favourable in the AP plane. For alignment in the lateral plane, the angulation between the axis of the proximal fragment of the fracture and the diaphyseal axis was measured, and values below 20° were considered appropriate. Medial cortical support (MCS) in the AP plane and anterior cortical support (ACS) in the lateral plane were analysed to evaluate fracture displacement. Neutral or positive states for MCS and ACS were considered appropriate [22, 23]. Examples of radiological examinations are shown in Figs. 1, 2, 3 and 4.

In addition to all the parameters mentioned, a scoring system based on our examination parameters generally accepted in the literature was developed to evaluate the surgical technique’s accuracy and overall success [20, 23–25]. Thus, the aim was to create a scoring model that can be used in clinical practice by determining the inclusive criteria for implant placement and reduction quality. The total score of the surgical criteria was named 'targeted surgical score (TSS) (0–8 points)', and it was planned to analyse the cumulative effect of the factors that play a role in the development of mechanical complications. The scoring system is structured as in Table 1.

**Table 1:** Targeted surgical score system for proximal femoral nailing

Targeted Surgical Score	Total 8 points
**Tip-apex distance**	**(0–2 points)**
Target: < 25 mm	+ 2 points
Acceptable: 25–30 mm	or + 1 point
Undesirable: ≥ 30 mm	or 0 point
**Lag screw quadrant**	**(0–2 points)**
Target: centre-centre or inferior-centre	+ 2 points
Undesirable: superior-posterior or superior-anterior	or 0 point
Acceptable: others	or + 1 point
**Fracture alignment**	**(0–2 points)**
Target AP alignment: neutral or < 10 degrees valgus	+ 1 point
Target lateral alignment: < 20 degrees angulation	+ 1 point
**Fracture displacement**	**(0–2 points)**
Target reduction on the AP: neutral or positive MCS	+ 1 point
Target reduction on the lateral: neutral or positive ACS	+ 1 point

### Statistical analysis

Data were analysed using IBM^®^ SPSS^®^ Statistics version 27.0.1 software. Descriptive statistics of continuous variables were expressed as mean, standard deviation, minimum, maximum and median values. Frequency analyses of categorical variables were performed, and percentages were reported. The normality distribution of continuous variables was analysed using the Shapiro–Wilk test. Pearson Chi-square test was used to compare categorical data between groups, and the Mann–Whitney U test was used to make pairwise comparisons of continuous variables—z-test analysed frequency differences of lag screw quadrants between the groups. Spearman correlation analysis was performed to determine the variables associated with complications. Multivariate logistic regression analyses were performed to examine the independent effects of the variables with a significant relationship in the correlation analysis on complication, and β-coefficients and Odds ratio (OR) values were analysed. Also, variance-induced factor (VIF) was analysed for multicollinearity control. Then, intergroup comparison and logistic regression analyses were performed for TSS and complications. The receiver operating characteristic (ROC) curve was constructed, and area under curve (AUC) and optimal threshold analyses (by Youden's J method) were performed. Likelihood ratio, Hosmer–Lemeshow and Pseudo R2 (by McFadden's method) results were analysed to evaluate model significance and fit. Clinical risk groups and corresponding TSS values were determined by comparing the complication probabilities predicted by the regression model with the actual complication rates. The significance limit was accepted as p < 0.05 in all statistical analyses.

## Results

This study included 586 patients, 48 of whom were in the complication group (8.2%) and 538 in the non-complication group (91.8%). Our study found no significant difference in patients' non-modifiable parameters between the groups. The detailed results of the groups in terms of demographic characteristics, fracture types and stability, proximal femur morphology, BMI and perioperative evaluation parameters are shown in Table 2.

**Table 2:** Results of the patients' unmodifiable parameters

Category	Parameter	Complication group (n = 48)	Non-complication group (n = 538)	p-Value
Demographics	Mean age (years)	79.25 ± 7.39	79.94 ± 7.55	0.44
	Age group 65–74 (%)	29.2% (n = 14)	26% (n = 140)	0.786
	Age group 75–84 (%)	45.8% (n = 22)	44.2% (n = 238)	
	Age group ≥ 85 (%)	25% (n = 12)	29.7% (n = 160)	
	Male (%)	27.1% (n = 13)	30.3% (n = 163)	0.642
	Female (%)	72.9% (n = 35)	69.7% (n = 375)	
	Fracture side right (%)	54.2% (n = 26)	53.2% (n = 286)	0.893
	Fracture side left (%)	45.8% (n = 22)	46.8% (n = 252)	
Fracture type	A1.2	33.3% (n = 16)	35.5% (n = 191)	0.994
	A2.2	33.3% (n = 16)	29.7% (n = 160)	
	A2.3	16.7% (n = 8)	19.9% (n = 107)	
	Others	16.7% (n = 8)	14.9% (n = 80)	
Fracture stability	Stable	33.3% (n = 16)	35.5% (n = 191)	0.783
	Unstable	66.7% (n = 32)	64.5% (n = 347)	
Bone morphology and BMI	CTI	0.48 ± 0.1	0.47 ± 0.09	0.318
	CFI	2.98 ± 0.62	2.91 ± 0.62	0.37
	BMI	25.25 ± 2.61	24.83 ± 2.94	0.348
Peri-operative parameters	ASA-II (%)	8.3% (n = 4)	15.4% (n = 83)	0.404
	ASA-III (%)	85.4% (n = 41)	79.6% (n = 428)	
	ASA-IV (%)	6.3% (n = 3)	5% (n = 27)	
	Spinal anaesthesia (%)	68.8% (n = 33)	75.5% (n = 406)	0.304
	General anaesthesia (%)	31.3% (n = 15)	24.5% (n = 132)	
	Waiting time (days)	4.0 ± 2.13	4.17 ± 1.81	0.512
	Operation time (mins)	91.56 ± 20.45	89.86 ± 22.23	0.644
	Discharge time (days)	4.13 ± 1.55	4.01 ± 1.47	0.691

### Radiological measurements and scoring criteria

There was no significant difference in the mean CDA on the fractured side (p = 0.089). However, when the difference between the fractured side and the intact hip was evaluated, there was a significant difference between the groups (p = 0.002). The fact that the mean difference was more negative indicates that the varus alignment was significantly higher in the complication group. All radiological parameters examined are shown in Table 3.

**Table 3:** Significant results of radiological evaluations

Parameter	Complication group (n = 48)	Non-complication group (n = 538)	p-Value
CDA (°)	130 ± 10.99 (100–153, median: 131.5)	132.95 ± 6.55 (108–160, median: 133)	0.089
CDA difference (°)	− 5.27 ± 9.8 (− 35 to + 18, median: − 4)	− 1.23 ± 6.05 (− 27 to + 20, median: 0)	**0.002**
Lateral angulation (°)	11.58 ± 6.70 (3–34, median: 11)	8.81 ± 4.95 (0–26, median: 9)	**0.013**
TAD (mm)	31.09 ± 11.22 (12.1–55.1, median: 30.3)	23.66 ± 9.46 (6.8–55.9, median: 22.1)	**< 0.001**
Lag quadrant distribution (%)	Superior-anterior (6.3%)	Superior-anterior (1.1%)	**0.006**
	Superior-posterior (25%)	Superior-posterior (4.6%)	**< 0.001**
	Centre-centre (18.8%)	Centre-centre (35.5%)	**0.019**
	Inferior-centre (10.4%)	Inferior-centre (26.2%)	**0.015**

Statistically significant results (p < 0.05) are shown in bold

**Fig. 5:**
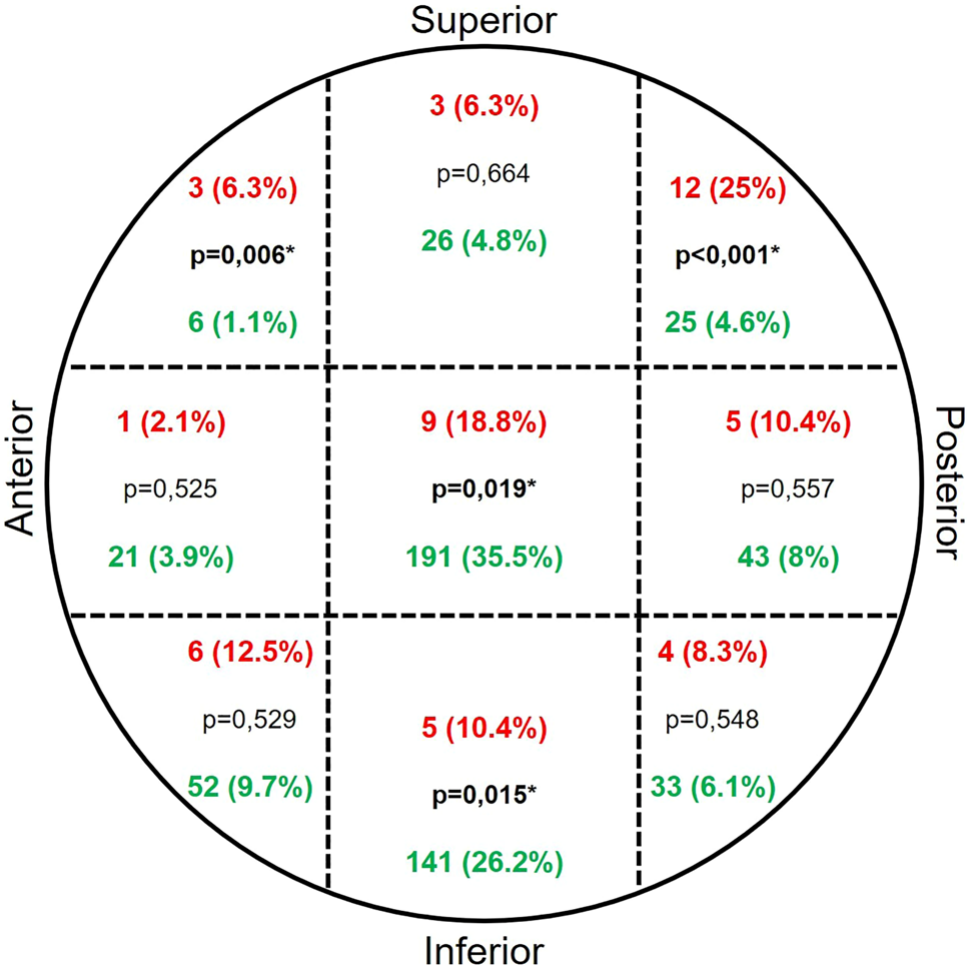
Number and distribution of patients with and without complications according to lag screw quadrants (complication group in red, uncomplicated in green). The p-value is from the z-test for comparison with distribution ratios

The frequency distribution of the groups in terms of the quadrant in which the lag screw was placed is shown in the Fig. 5. The frequency of patients with complications in the superior anterior and superior posterior quadrants was significantly higher than those without complications. The frequency of patients with complications in the centre centre and inferior centre quadrants was significantly lower than those without complications (Fig. 5, Table 3).

The detailed results of the analyses of the sub-scoring criteria of the TSS system are presented in Table 4. According to these results, it was determined that all criteria showed a significant difference and a weak negative correlation on complication. Additionally, the results of the multivariate logistic regression analysis performed to investigate the independent effect of each scoring criterion on complication are as in Table 5. The criteria with the highest independent effect on complications were the lag quadrant and AP-MCS scores, and the lowest were AP and lateral alignment scores. The multicollinearity problem was within acceptable limits for all criteria (VIF < 5).

**Table 4:** Results of sub-scoring criteria and correlation analysis

Parameter	Complication group (n = 48)	Non-complication group (n = 538)	Correlation coefficient (r)	p-Value
TAD score	0.79 ± 0.92	1.41 ± 0.85	− 0.193	**< 0.001**
Lag quadrant score	0.98 ± 0.79	1.56 ± 0.6	− 0.221	**< 0.001**
AP alignment score	0.42 ± 0.5	0.57 ± 0.5	− 0.083	**0.045**
Lateral alignment score	0.9 ± 0.31	0.98 ± 0.13	− 0.157	**< 0.001**
AP-MCS score	0.42 ± 0.5	0.7 ± 0.46	− 0.168	**< 0.001**
Lateral-ACS score	0.56 ± 0.5	0.91 ± 0.29	− 0.296	**< 0.001**

Statistically significant results (p < 0.05) are shown in bold

**Table 5:** Multivariate regression analysis results of sub-scoring criteria

Parameter	β (coef.)	SE	Odds R. [CI 95%]	p-Value	VIF
TAD Score	− 0.738	0.166	0.478 [0.345–0.661]	**< 0.001**	2.1
Lag quadrant score	− 1.204	0.215	0.300 [0.197–0.457]	**< 0.001**	3.5
AP alignment score	− 0.606	0.305	0.546 [0.300–0.993]	**0.047**	1.8
Lateral alignment score	− 0.585	0.199	0.557 [0.377–0.823]	**0.003**	2.7
AP-MCS score	− 1.196	0.308	0.302 [0.165–0.552]	**< 0.001**	2.9
Lateral-ACS score	− 1.111	0.188	0.329 [0.228–0.475]	**< 0.001**	3.2

Statistically significant results (p < 0.05) are shown in bold

### Targeted surgical score statistics

The mean TSS was 4.06 ± 2.22 (min–max: 0–8, median: 4.5) in the complication group and 6.14 ± 1.56 (min–max: 0–8, median: 6) in the uncomplicated group. TSS was significantly higher in the uncomplicated group (p < 0.001). In addition, in logistic regression analysis between TSS and complications, the β-coefficient was − 0.597 (SE 0.085, 95% CI − 0.764 to − 0.430), OR 0.551 (95% CI 0.466–0.651) and p < 0.001. These results suggest that a higher TSS is associated with a lower likelihood of complications. The OR value obtained suggests that a one-unit increase in TSS reduces the probability of developing complications by 44.9%.

**Fig. 6:**
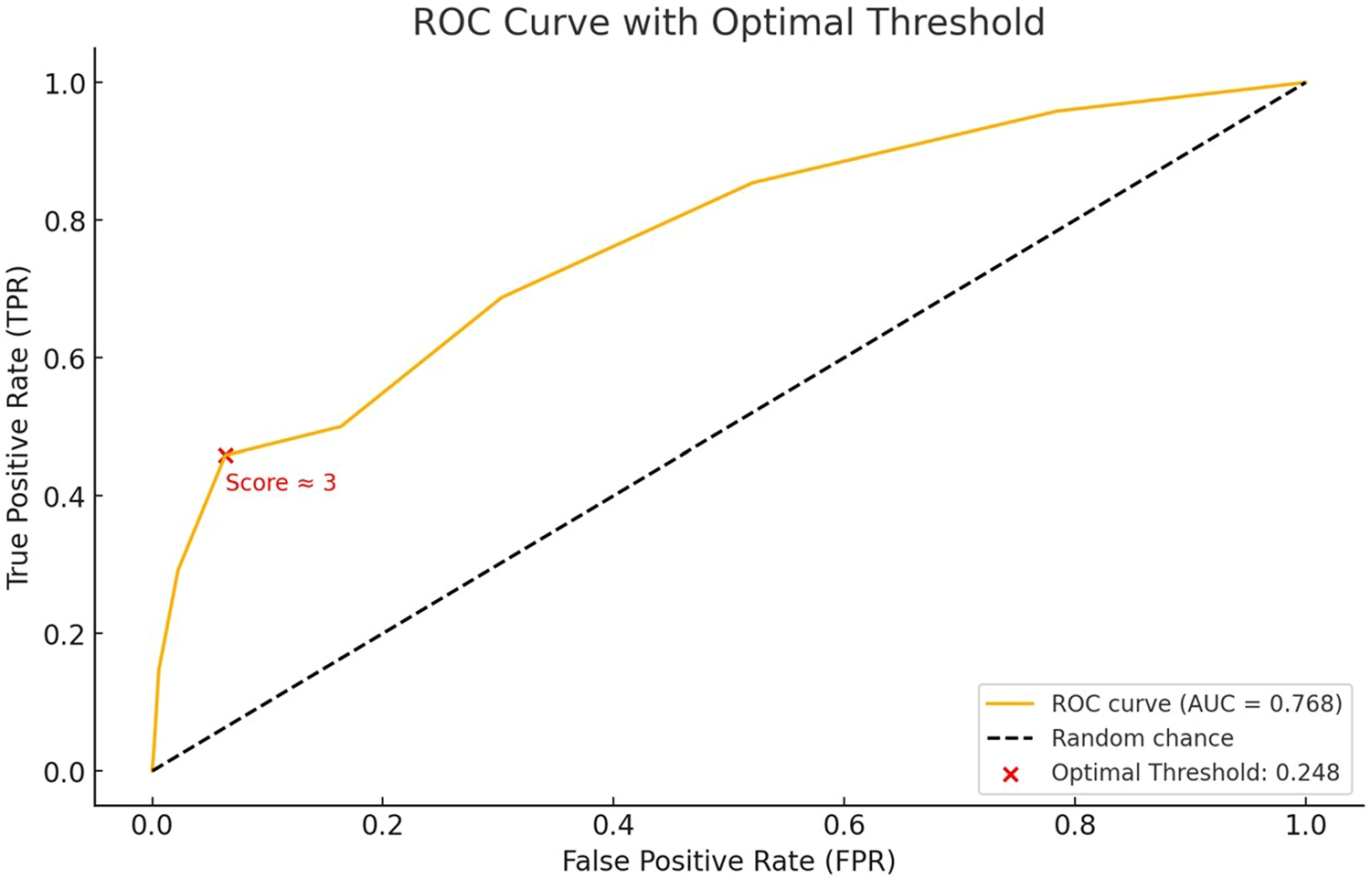
ROC curve analysis for the performance of the Targeted Surgical Score in discriminating complications (Youden's J statistic calculated the optimal threshold value and the corresponding TSS)

ROC analysis to evaluate the complication prediction performance of TSS, and the AUC value was calculated as 0.768. This value suggested that the model has a moderate-to-good predictive ability for complications (Fig. 6). According to Youden's J statistic, the optimal threshold value was 0.248. The TSS corresponding to the threshold value was calculated as 3 points. The risk of complications can be considered higher when TSS is 3 points or less (sensitivity: 45.8%, specificity: 93.7%, accuracy: 89.76%). The likelihood ratio test p-value of < 0.001 was conducted to assess the overall model fit, indicating that including the TSS variable significantly improved the model's predictive capability for complications. The Hosmer–Lemeshow test produced a p-value of 0.243, suggesting no significant difference between observed and expected results within the TSS model, affirming its goodness of fit. The McFadden R^2^ value was calculated as 0.17, reflecting a moderate-good explanatory power of the TSS variable for complications (McFadden R^2^ for logistic regression models, 0.20 to 0.40 are considered very good).

**Fig. 7:**
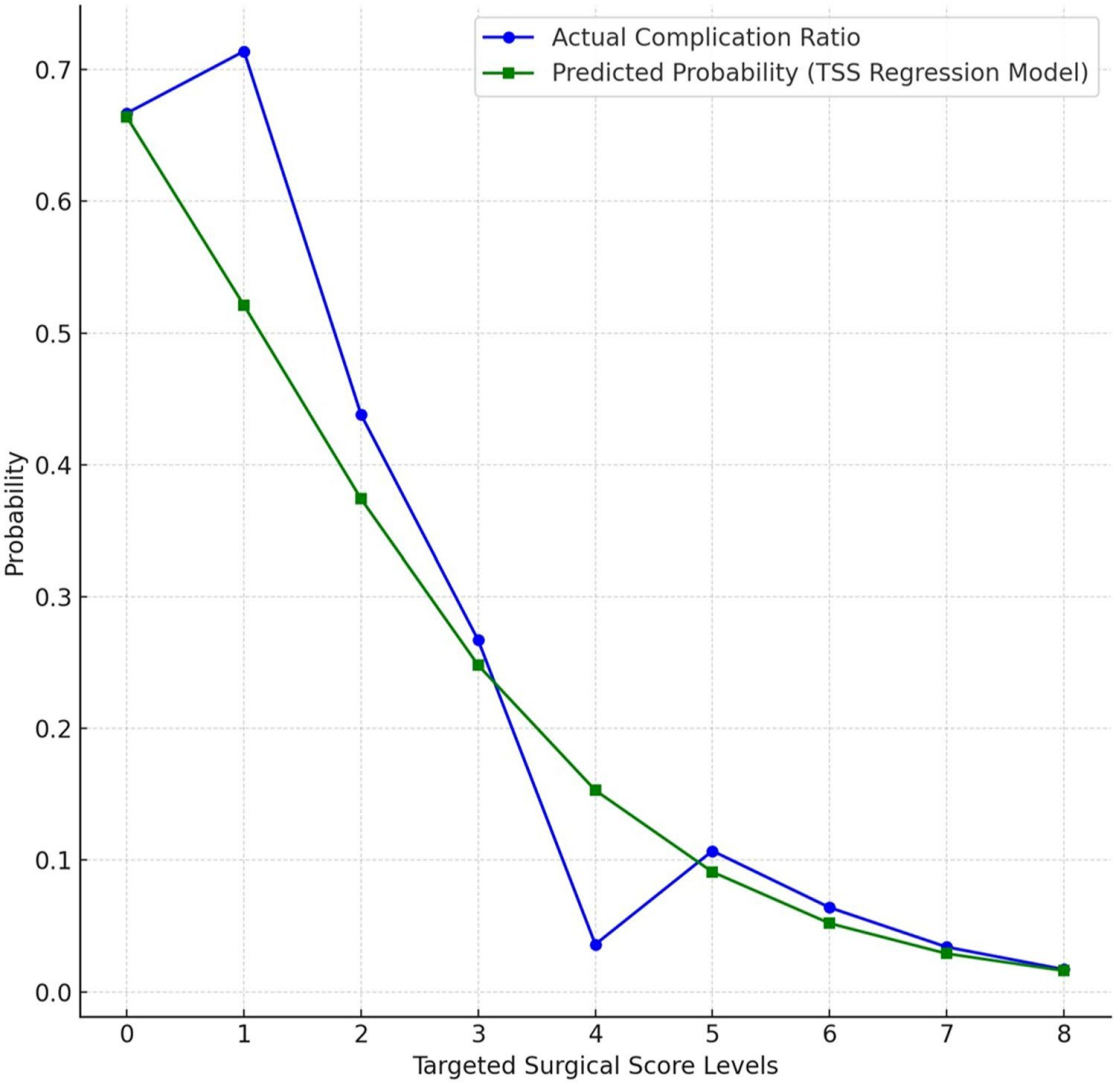
Graph of actual and predicted complication rates according to targeted surgical score levels

The comparison of the complication probabilities calculated for all scores between TSS 0 and 8 through the regression model and the actual complication rates corresponding to the scores in the data set are shown in Fig. 7. Upon reviewing the table and graph, it is evident that the TSS model accurately identifies complication probabilities with minimal deviation and high precision for scores of 5 and above. Based on all statistical analyses conducted between TSS and complications, clinical risk categories and corresponding TSS scores were defined in Table 6.

**Table 6:** Clinical risk categories to targeted surgical score

Clinical risk category	Complication probability (%)	TSS
Very low risk	< 5	7 and 8
Low risk	5–10	5 and 6
Moderate risk	10–50	2, 3, and 4
High risk	> 50	0 and 1

## Discussion

Our study comprehensively analysed the surgical factors that could contribute to mechanical complications in treating femoral intertrochanteric fractures with PFN in elderly patients. Our findings indicate that implant placement techniques and surgical parameters may significantly influence complication rates, whereas non-modifiable patient characteristics within our evaluation parameters appear to have a limited impact. While these results align broadly with existing literature, our scoring system suggests an innovative perspective for predicting and mitigating complications.

There is a wide variation in the reported complication rates after treatment of femoral trochanteric fractures with PFN in the literature. Our study found a mechanical complication rate of 8.2%, consistent with rates ranging from 5 to 20% in the literature [24, 26, 27]. A recent meta-analysis reported that overall complication rates associated with the use of PFN were between 10–15%, and these rates were associated with screw migration, cutout, varus collapse, and implant failures [28]. Our complications were cutout, screw migration and implant failure, similar to the literature that coincides with the surgical technique errors and implant placement problems described in the existing literature. The consistency of our complication rates with previous studies suggests that our findings align with existing literature.

No significant difference was observed between the groups with and without complications regarding demographic characteristics such as age, age group, gender and side. Similarly to our results, the lack of effect of age on the risk of complications was previously reported earlier [29, 30]. The most common fracture types according to the AO/OTA classification were A1.2 and A2.2, and no significant difference was found between the groups in terms of fracture types and fracture stability. Although the literature has reported that unstable fractures may increase the risk of mechanical complications, this did not make a significant difference in our study [24, 27]. This situation may be due to fewer patients in the complication group. Expanding the number of patients with complications in multicentre studies may provide a more accurate interpretation of the results.

Although it has been previously demonstrated that high ASA scores may adversely affect general results, there was no significant difference between the groups in our study regarding preoperative ASA scores, which evaluate the patient's general health conditions and comorbidities [24, 29]. When the effect of anaesthesia type on the development of complications was examined, no significant difference was found between the groups. In both groups, spinal anaesthesia was preferred more frequently than general anaesthesia. The patients'waiting, operation, and discharge times were similar in both groups.

In our study, BMI values were not significantly associated with mechanical complications. This result is consistent with the study of Ciufo et al*.*, who stated that high BMI may increase the risk of cutout and implant migration. However, this relationship was not statistically significant [10]. On the other hand, Soucanye de Landevoisin et al*.* reported that low BMI was associated with surgical technique accuracy rather than complication development [29]. Similarly, no significant difference was found between the groups for CTI and CFI values, which we analysed regarding morphology and bone quality of the proximal femur. Like our study, Bozgeyik et al*.* emphasised that CTI and CFI do not directly correlate with complications [30]. In our retrospective study, direct bone quality measurement data, such as bone mineral density (BMD) or CT-based Hounsfield Unit (HU) analysis, were unavailable for all patients. Thus, they were not incorporated into the analysis. As an alternative, age groups, CTI, and CFI parameters were analysed to provide insight into overall bone quality and osteoporosis. However, these parameters did not show significant differences. Despite this, they may not fully capture the impact of osteoporosis and bone quality on complications. Therefore, future studies should incorporate objective bone quality assessments to address this limitation.

In our study, there was no difference between the groups regarding unmodifiable factors related to the patient, such as demographic characteristics, fracture type and stability, perioperative anaesthesia and surgical processes, BMI, and bone morphology. The similar results of the groups allowed intergroup standardisation in evaluating surgeon-related parameters. This could emphasise the importance of the differences in the modifiable factors related to surgical practice.

### Importance of surgery-related parameters

Tip-apex distance is one of the critical parameters for implant-related complications in the surgical treatment of femoral trochanteric fractures. In our study, the mean TAD was 31.09 ± 11.22 mm in the group with complications and 23.66 ± 9.46 mm in the group without complications, and this difference was statistically significant (p < 0.001). TAD, first defined by Baumgaertner et al*.*, represents a threshold value below which the risk of the cutout is significantly decreased when it is below 25 mm. In comparison, the risk of failure prominently increases when it is 30 mm and above [20]. Many studies have supported the accuracy of these cut-off values [24, 31–33]. Systematic reviews also emphasise the decisive role of TAD in the risk of complications [28, 34]. In addition, Caruso et al*.* stated that new parameters such as calcar-referenced TAD (CalTAD) play a vital role in complication prediction and that its use in combination with conventional TAD will provide a more reliable risk analysis [35, 36]. Our findings are consistent with many previous studies and reinforce that maintaining appropriate TAD values helps reduce complications.

According to the Cleveland-Bosworth classification, central-central or inferior-central positioning of the lag screw has been defined as the ideal position to increase mechanical stability and reduce the risk of the cutout [37, 38]. In our study, it was found that the lag screw was frequently located in the superior anterior (6.3%, p = 0.006) and superior posterior (25%, p < 0.001) quadrants in the complication group. In comparison, it was located in the central-central (35.5%, p = 0.019) and inferior-central (26.2%, p = 0.015) quadrants in the group without complications (Table 3, Fig. 5) Erinç and Özdemir reported that peripherally located screws were the factor that increased the risk of cutout the most [39]. Tosounidis et al*.* showed that the inferior-central position maximises axial and torsional stability, and calcar-supported placement significantly reduces the risk of mechanical failure [31]. Herman et al*.* stated that superior placements reduce the load-bearing capacity and increase the risk of screw migration [40]. Furthermore, Caruso et al*.* and Yoo et al*.* reported that central and inferior placements are safer, whereas superior or posterior placements increase the risk of complications [26, 35]. Our findings, consistent with previous studies, suggest that superior-anterior and superior-posterior placements could increase the risk of complications, whereas central and inferior placements appear to enhance mechanical stability. Ensuring proper screw placement according to the Cleveland-Bosworth classification with fluoroscopic control during surgery seems to be an essential factor in reducing complication rates.

The quality of fracture reduction is critical for reducing complications. Recently, cortical contact and alignment between the fracture fragments have been emphasised as more important for secondary stability. Studies have shown that assessments of cortical alignment and contact in AP and lateral planes are more valuable than the amount of displacement [23, 41, 42]. Therefore, we preferred using the Chang et al*.* method to evaluate the reduction quality in our study. Chang et al*.* reported that MCS reduces mechanical complications and stabilises neck-shaft angle and femoral neck length [22, 24]. Mao et al*.* emphasised that MCS limits implant slippage and increases stability and stated that its absence may lead to varus deformity [23]. Kristan et al*.* and Itou et al*.* showed that MCS is critical in preventing complications such as cutouts and implant failure [41, 43]. Shon et al*.* stated that lack of MCS causes varus malposition and loss of rotational stability [44]. Similarly, Chang et al*.* stated that ACS in the lateral plane improves the reduction quality and reduces implant failures [22, 24]. Yoon et al*.* stated that ACS is a critical element for optimal reduction, and the continuity of the anterior cortical line should be ensured [7]. Kristan et al*.* reported that a lack of ACS leads to mechanical complications, and Itou et al*.* reported that negative ACS increases postoperative complication rates [41, 43]. Herman et al*.* emphasised that ACS limits implant movement by providing secondary stability [40]. Our study revealed that mechanical complication rates were significantly lower when the literature provided MCS and ACS. In line with these findings, intraoperative optimisation of MCS and ACS in surgical applications is a strategic approach to prevent mechanical complications.

Providing an appropriate fracture alignment in both AP and lateral planes during surgery is another critical factor in the success of the treatment without complications. Turgut et al*.* emphasised that the risk of cutout increased in patients with CDA < 130° and that mild valgus reduction improves stability [13]. Similarly, different studies have reported that mild valgus alignment favours implant stability, whereas varus alignment increases the risk of failure [10, 32, 45]. Our study determined that CDA values in the complication group deviated more from neutral and shifted to a varus position compared to the intact side. This finding is consistent with the existing data in the literature. Angulation in the lateral plane is as essential as AP alignment. Chang et al*.* stated that angulation < 20° in the lateral plane is a criterion for the reduction to be considered 'good'[24]. Mao et al*.* stated lateral angulation negatively affects mechanical stability by increasing implant migration and varus deformity [23]. Babhulkar et al*.* emphasised that excessive deviations in the lateral plane increase the risk of varus alignment and lead to implant failures [46]. In our study, complication rates were higher in cases with lateral angulation > 20°. According to the literature and our findings, keeping the CDA in neutral or slightly valgus position in the AP plane and the angulation in the lateral plane below 20° are the points to mitigate complications.

### Our radiological scoring model "Targeted Surgical Score"

Targeted Surgical Score, which we designed to assess the risk of mechanical complications and support preventive strategies, was evaluated in an elderly patient population undergoing PFN This study explores the association between modifiable surgical factors and mechanical complications in a systematic manner. It proposes a scoring model that estimates their cumulative impact.

The mean TSS score of 4.06 ± 2.22 points in the complication group and 6.14 ± 1.56 points in the uncomplicated group (p < 0.001), indicating that this scoring system has potential for risk stratification. According to logistic regression analysis, each unit increase in the TSS was associated with a 44.9% reduction in complication risk (OR 0.551; p < 0.001). These findings suggest that the TSS reflects the quality of surgical technique and reduction, and higher scores are associated with a lower risk of mechanical complications. Key factors influencing complication risk included the lag quadrant score (OR 0.300), AP-MCS score (OR 0.302), Lateral-ACS score (OR 0.329), and TAD score (OR 0.478), whereas the effect of AP and lateral alignment scores was limited. These findings suggest that placing the lag screw in the appropriate quadrant, ensuring medial and anterior cortical supports during fracture reduction, and maintaining proper TAD values are essential factors in minimising the risk of mechanical complications.

### Clinical applicability

A fully comprehensive intraoperative application of the TSS model based on correctable surgical factors may not always be feasible in clinical practice. However, it may enhance surgeons'awareness and encourage a greater focus on critical factors that require attention. Thus, TSS may minimise mechanical complications by guiding intraoperative decision-making and promoting surgical optimisation in clinical practice. In the future, integrating software tools into intraoperative imaging systems could facilitate real-time measurements and optimise surgery-dependent correctable parameters.

Beyond its potential intraoperative utility in mitigating complications, TSS may also classify patients into risk groups based on detailed early postoperative radiographs [Table 6]. This classification may allow for patient-specific follow-up and rehabilitation protocols, enabling closer monitoring and a more tailored rehabilitation program for high-risk patients. In addition, it may assist physicians in providing patients with preliminary information about their current complication risk and the potential need for secondary surgery.

### Strengths and improvable aspects

The major value-adding of our study was suggesting a comprehensive surgical scoring system. While the existing methods in the literature usually focus on isolated factors, our study has considered multiple surgical parameters with a holistic approach. The TSS system, which integrates the cumulative effect of multiple surgical parameters, may provide a helpful clinical usability and scientific exploration framework.

Unlike the existing systems that analyse individual parameters, such as only TAD or Cleveland–Bosworth classification, the TSS considers the influence of surgical technique and reduction quality on complications risk in a multidimensional manner. All sub-scoring criteria of the TSS have been significantly associated with complications in both our study and the literature. Although the impact of each parameter on complications varies, we preferred developing an equally weighted scoring preliminary model to enhance simplicity and clinical usability.

The AUC value of 0.768 from the ROC analysis suggest that the model demonstrates moderate-to-good predictive performance in identifying complications (Fig. 6). Moreover, the predictive performance of the TSS aligns statistically with actual complication rates. In particular, a score level ≥ 5 was associated with lower complication rates, demonstrating high specificity, accuracy, and consistency (Fig. 7). Optimal threshold analysis showed that at a TSS level of approximately 3 points, the sensitivity was 45.8%, specificity was 93.7%, and accuracy was 89.6% (Fig. 6). These results indicate that while the TSS model has limited sensitivity in detecting complications, it exhibits high specificity in identifying uncomplicated cases. Thus, higher scores may serve as a valuable indicator for minimising complication risk in clinical practice. In addition, the scoring model could categorise patients with high, moderate, low and very low risk, which may guide clinical usage (Table 6).

Among studies with similar methodologies, Kulakoğlu et al*.* proposed a new scoring system to predict the cutout risk [47]. Based on their case series analysis, they developed a weighted scoring system by identifying the parameters most associated with cutout. Their model incorporated patient-dependent, non-modifiable factors such as gender and surgically modifiable parameters, including TAD, CalTAD, and reduction quality. Instead of relying solely on our study’s findings, we preferred developing a scoring system based on widely accepted parameters in the literature, supported by multiple studies and meta-analyses. Additionally, we thoroughly examined whether non-modifiable patient factors significantly differed between groups to prevent potential misinterpretations. Thus, our surgery-related parameters, broadly consistent with the literature, were incorporated as scoring criteria in the TSS. This approach may offer an advantage for the generalisability of the results and the clinical usability of the proposed scoring system. However, all the criteria in our scoring model were assigned equal weights. Adjusting the weight each criterion based on its independent impact on complications may enhance the scoring system’s predictive performance. Future studies should aim to refine and develop the TSS model by implementing a weighted scoring system based on logistic regression coefficients.

### Limitations

There are several limitations to this study. Its design is based on a single-centre retrospective analysis, which may limit the generalisability of the results and increase the risk of missing data. Prospective, multicentre studies should be conducted to validate the TSS system externally. This study does not include measures such as BMD or CT-based HU, which could provide a more objective assessment of bone quality and its impact on complications. Additionally, the scoring criteria were not weighted according to their independent effects on complications. Additional factors not considered in this study, such as surgeon experience, implant design variations, and patient adherence to rehabilitation, represent potential confounders that may have influenced the results. Future research should be designed to address these limitations.

## Conclusion

Our findings suggest that a higher TSS may be linked to a lower risk of mechanical complications. Based on our scoring system, a TSS of one or lower may indicate a high risk of complications, while a score of five or higher may help mitigate these risks. This novel system provides a structured approach for estimating complication risk and may aid intraoperative decision-making. Key intraoperative factors include achieving medial and anterior cortical support during fracture reduction and placing the lag screw in the appropriate quadrant with an optimal tip-apex distance. The TSS may serve as a valuable tool for guiding surgical decisions and identifying patients at high risk for postoperative complications, facilitating closer follow-up and tailored rehabilitation.

In conclusion, the TSS may be a promising guide for predicting and reducing the risk of mechanical complications following PFN treatment of elderly femoral trochanteric fractures. However, multicentre prospective studies with larger patient cohorts are needed to validate and generalise the model. Further research should focus on integrating objective bone quality measurements and refining the weighting of scoring parameters to enhance clinical applicability.

## Data Availability

The datasets used and/or analysed during the current study are available from the corresponding author on reasonable request. No datasets were generated or analysed during the current study.

## Abbreviations

ACS: Anterior cortical support
AO/OTA: Arbeitsgemeinschaft für Osteosynthesefragen/Orthopaedic Trauma Association
AP: Anteroposterior
ASA: American Society of Anesthesiologists
AUC: Area under curve
BMI: Body mass index
CalTAD: Calcar-referenced tip-apex distance
CDA: Collo-diaphyseal angle
CFI: Canal flare index
CI: Confidence interval
CTI: Cortical thickness index
MCS: Medial cortical support
OR: Odds ratio
PFN: Proximal femoral nail(ing)
ROC: Receiver operating characteristic
SE: Standard error
TAD: Tip-apex distance
TSS: Targeted surgical score
VIF: Variance-induced factor
